# In silico screening of neurokinin receptor antagonists as a therapeutic strategy for neuroinflammation in Alzheimer’s disease

**DOI:** 10.1007/s11030-021-10276-6

**Published:** 2021-07-31

**Authors:** Sairaj Satarker, Swastika Maity, Jayesh Mudgal, Madhavan Nampoothiri

**Affiliations:** Department of Pharmacology, Manipal College of Pharmaceutical Sciences, Manipal Academy of Higher Education, Manipal, Karnataka 576104 India

**Keywords:** Alzheimer’s disease, Substance P, Neurokinin 1 receptor, Molecular dynamics, MM-GBSA, l-NAT

## Abstract

Neuroinflammation is one of the detrimental factors leading to neurodegeneration in Alzheimer’s disease (AD) and other neurodegenerative disorders. The activation of microglial neurokinin 1 receptor (NK1R) by substance P (SP) enhances neuroinflammation which is mediated through pro-inflammatory pathways involving NFkB, ERK1/2, and P38 and thus projects the scope and importance of NK1R inhibitors. Emphasizing the inhibitory role of *N* Acetyl l Tryptophan (l-NAT) on NK1R, this is the first in silico screening of l-NAT mediated NK1R antagonism. In addition, FDA- approved ligands were screened for their potential NK1R antagonism. The l-NAT was docked in XP (Extra Precision) mode while FDA-approved ligands were screened in HTVS (High Throughput Virtual Screening), SP (Standard Precision), and XP mode onto NK1R (PDB:6HLO). The l-NAT and top 3 compounds FDA-approved ligands were subjected to molecular dynamics (MD) studies of 100 ns simulation time. The XP docking of l-NAT, indacaterol, modafinil and alosetron showed good docking scores. Their 100 ns MD showed brief protein–ligand interactions with an acceptable root mean square deviation. The protein–ligand contacts depicted pi-pi stacking, pi-cation, hydrogen bonds, and water bridges with the amino acids necessary for NK1R inhibition. The variable colour band intensities on the protein–ligand contact map indicated their binding strength with amino acids. The molecular mechanics/generalized born surface area (MM-GBSA) scores suggested favourable binding free energy of the complexes. Thus, our study predicted the ability of l-NAT, indacaterol, modafinil, and alosetron as capable NK1R inhibitors that can aid to curb neuroinflammation in conditions of AD which could be further ascertained in subsequent studies.

**Graphic Abstract:**

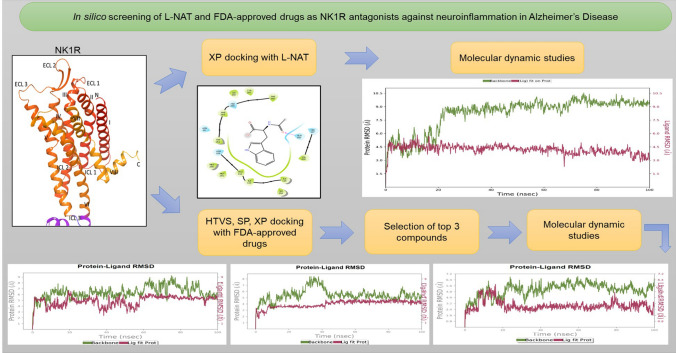

## Introduction

Neuroinflammation is a vital player in central nervous system (CNS) disorders, be it acute or chronic conditions. The immune response of CNS triggers cascading events that lead to the development and progression of neurodegenerative diseases like Alzheimer’s Disease (AD) [1]. Along with the presence of amyloid-beta (Aβ) plaques and neurofibrillary tangles (NFT), the contribution of glial cells like astrocytes and microglia have marked their significance in the release of inflammatory cytokines promoting neuroinflammation [2].

In the pathological conditions of AD, the Aβ oligomers provoke noxious stimuli leading to the release of Substance P (SP) that binds to NK1R leading to its activation followed by the release of inflammatory cytokines [3] as seen in Fig. 2. Repetitive microglial self-activation can lead to the reactivation of its own and neighbouring microglia, followed by the release of more inflammatory mediators and ultimately progress to neuroinflammation, oxidative stress and apoptosis [4].

SP and neurokinin receptor systems are known to be involved in neuroinflammation [5, 6]. Amongst the various receptors present on astrocytes and microglia [7, 8], microglial Neurokinin 1 receptor (NK1R) are known to be associated with inflammatory pathways [9]. SP upon synthesis in the nerve cell bodies of the corpus striatum [10] is released and rapidly binds to NK1R on the same or neighbouring cells facilitating neuroinflammation [11]. The SP has the highest affinity for Neurokinin 1 Receptor (NK1R) and is found in the olfactory regions, cerebral cortex, hippocampal formation, basal ganglia, amygdala, etc. in the CNS [12].

**Fig. 1:**
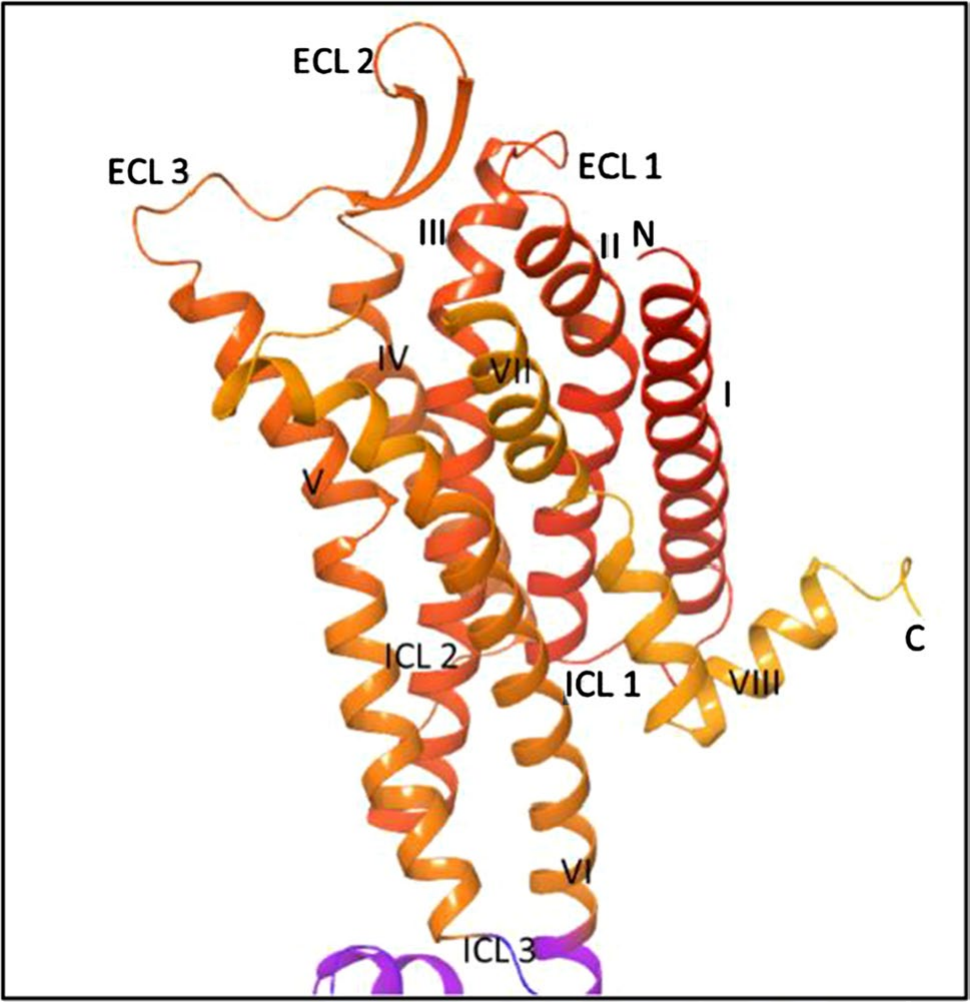
3D Structure of NK1R

The NK1R belongs to class I Rhodopsin like G-Protein Coupled Receptors (GPCR) having seven transmembrane helices with extracellular amino-terminal and intracellular carboxy-terminal and sometimes an 8th helix is also present parallel to the membrane as shown in Fig. 1. ECL 2 joins to form a β pleated hairpin-like structure that lies between the ECL II and third helix. The neurokinins contain a carboxy-terminal that is known to interact with the receptors, while amino acid sequence terminal codes for receptor specificity [13]. The ICL3 is responsible for G protein interactions and ELC2 and ELC3 are responsible for agonist or antagonist binding [14, 15]. Therefore, these aspects are essential to understand the binding characteristics of the NK1R to develop better candidates for its inhibition.

**Fig. 2:**
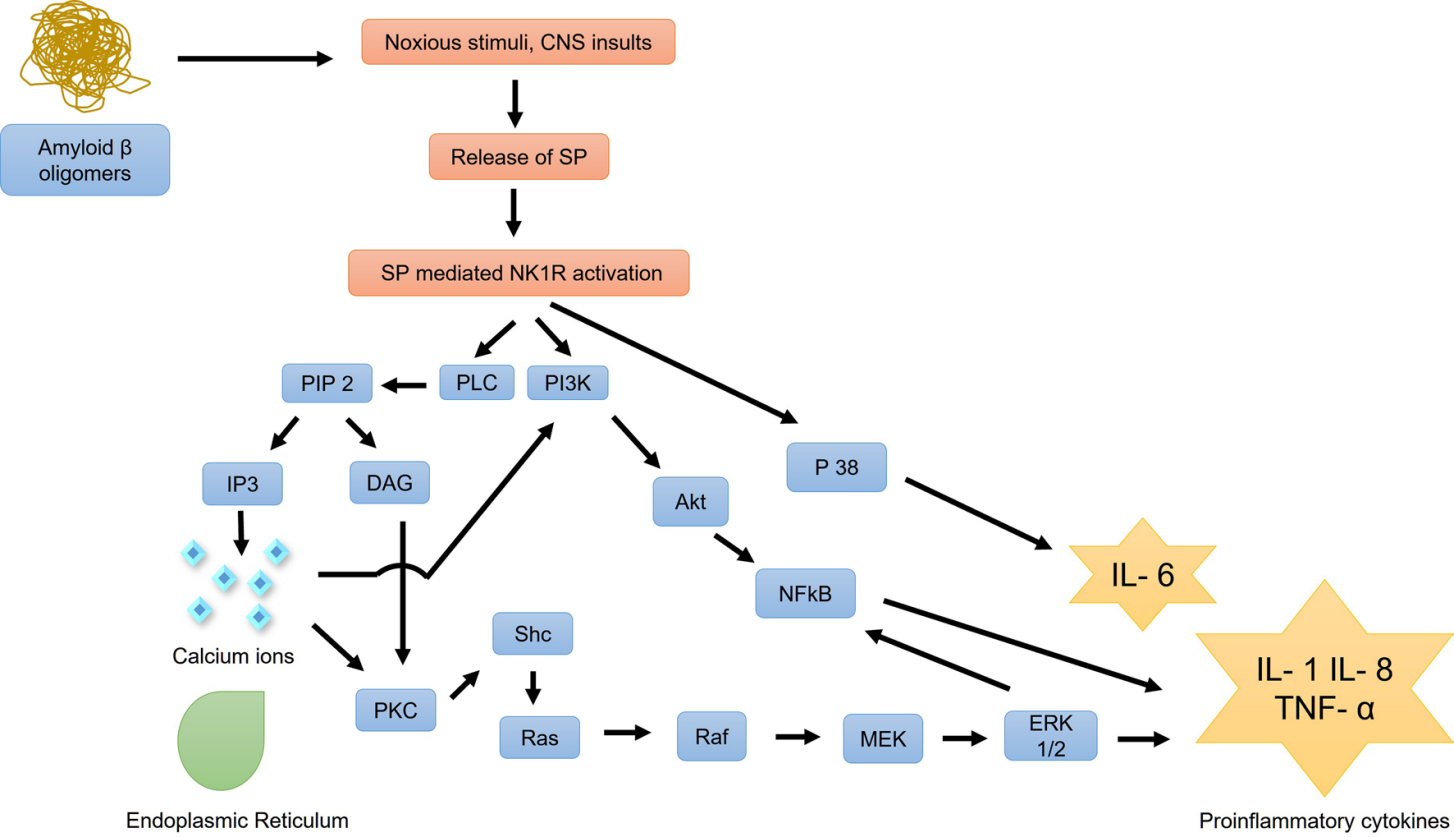
Activation of NK1R receptor by SP leading to the unravelling of inflammatory pathways Phosphatidylinositol 4,5-bisphosphate (PIP_2_), Phospholipase C (PLC), Inositol 1,4,5-triphosphate (IP_3_), Diacylglycerol (DAG), Protein Kinase C (PKC), Src homology 2 domain-containing transforming protein (Shc) Extracellular signal-regulated kinase (ERK), Phosphatidylinositol-3-kinase (PI3K), Protein Kinase B (AKT), Interleukin -6 (IL-6), Tissue Necrosis Factor (TNF-α), Nuclear Factor Kappa B (NFκB)

The development of new drug candidates consumes an ample amount of time and resources due to which drug repositioning or drug repurposing has gained a significant amount of importance in today’s field of drug discovery. The repurposing of the drugs approved by US-FDA (United States Food and Drug Administration) provides a quick method for discovering newer possible candidates for targets.

NK1R antagonists like aprepitant have been able to reduce the actions of SP and this inhibition may prove beneficial in CNS disorders [16]. Aprepitant has been approved to treat Cancer Induced Nausea and Vomiting (CINV) [17] in CNS but due to various side effects like diarrhoea, abdominal pain, leukopenia, indigestion, weakness, etc. [18] its use is limited. Its co-administration with pimozide leads to increased levels of pimozide causing QT prolongation [19]. Owing to its BBB permeability, it was also subjected to clinical trials to treat depression but failed at Phase III because of improper understanding of receptor engagement and output response and inadequate data of the highest dose that could be tolerated [20]. The effectiveness of aprepitant is also not justified in neuroinflammation and novel and safer therapeutic approaches are necessary.

The NK1R antagonist, l-NAT binds to NK1R with more affinity than *N* Acetyl d tryptophan (d-NAT) as the carboxyl group of the earlier lies in the plane while that of the latter lies out of a plane [21]. Recently in our laboratory, the role of NK1R antagonist l-NAT was evaluated in vivo that showed enhancement in memory in an aluminium chloride-induced rat model of dementia [22].

Therefore, our current research work emphasizes the role of *N* Acetyl l Tryptophan (l-NAT) along with FDA-approved ligands as NK1R antagonists that may prove an essential candidate in curbing neuroinflammation that could aid in AD therapeutics via the NK1R system. To the best of our knowledge, this is the first study exploring the in silico potential of l-NAT as an NK1R antagonist.

## Materials and methods

All the studies were carried out using Maestro software version 11.8 run through Schrodinger Suite 2020. To run the application a computer equipped with Linux Ubuntu operating system with built-in Haswell graphics, 4 GB RAM, and Intel Core i3 processor was used to perform all computer-aided simulation studies like docking studies, virtual screening, MM-GBSA, and molecular dynamic studies.

### Identification of crystal structure

The crystal structures of NK1R were scrutinized from the Protein Data Bank (PDB) and one structure (PDB ID: 6HLO) co-crystallized with its antagonist aprepitant was selected [23]. Among the available structures of NK1R in PDB, the structure with PDB ID: 6HLO was selected as it was bound with antagonist aprepitant that would help in indicating antagonist binding sites required for the study and it was having the best possible resolution of 2.40 Å compared to other PDB codes for NK1R. The 2D structure of *N* Acetyl l-Tryptophan (l-NAT) was drawn using the 2D sketcher module of Schrodinger Maestro. The FDA-approved ligands were obtained from Drug Bank (www.drugbank.com).

### Preparation of protein structure

The processing of NK1R structure 6HLO.pdb was done using the ‘Protein Preparation’ module. This module identifies and eliminates any defects in the structure, incorporates hydrogen atoms, allocates bond orders, tautomerization, and even ionization states, and enables network optimization of the hydrogen bonding. First, the structure was pre-processed whereby the missing loops were filled, water molecules were removed, hydrogen bonds were added, missing side chains and loops were filled using the PRIME function, as the downloaded PDB structures may lack atoms, charges, side chains, bond orders, etc. The structure was freed from additionally bound ligands. The pH plays an important role in the protonation states of ligands and residues and helps in simulating the exact experimental conditions. In our experimental setup, the PROPKA pH was set to 7.0. The addition of hydrogen bonds or filling missing sidechains can create issues due to which restraint minimization was carried out using OPLS3e force field at an RMSD of 0.3 Å where hydrogens and heavy atoms were minimized through harmonic penalty constraints.

### Preparation of ligand

l-NAT was our primary ligand of interest along with other FDA-approved drugs. The ligands were processed using the ‘LigPrep’ module of the Schrodinger Suite. The energy minimization was performed using the OPLS3e force field. The possible ionization states were generated at a pH of 7 ± 2 and chirality were determined from its 3D structure.

### Identification of additional binding sites

The protein structure (PDB ID: 6HLO) downloaded from PDB was co-crystallized to an antagonist, which could indicate its active binding site. To confirm this, we mapped additional druggable sites available on the protein using the SiteMap module of Schrodinger Suite and ranked them based on the site scores and druggability scores. The SiteMap module helps in locating possible binding sites and predict how druggable these sites can be. The site score obtained by this module can help in assessing the ability of the site in binding through hydrogen bond donors, hydrogen bond acceptors, and even the ability to bind to metals.

### Generation of receptor grid

Using the receptor grid generation module in Maestro Schrodinger, a grid was generated along with the binding site of the protein by choosing the atoms of the site obtained through a site map. Sometimes the receptors tend to shift their conformations upon binding for e.g. a change in its side chain conformation or change in the location of its backbone or even a change in its loop conformation. Therefore, it is essential to create a grid on which the structure and the properties of the receptor can be established so that the generated ligand poses can be accurately scored. First, the receptor structure was selected as the system considers only the selected region as a receptor to be used in further steps. Following this step, by selecting entry mode, the atoms of the sites generated by SiteMap were selected. Then the van der Waals radii for receptor atoms were scaled at a scaling factor of 1 at a partial charge cut-off of less than 0.25. An enclosed box was created that formed a grid at the centre of the selected residue of ligand length ≤ 20 Å.

### Docking of l-NAT and FDA approved ligands with NK1R

Based on the grids generated by the Glide Grid module, the ligand l-NAT was docked to the selected site using the Glide Dock module in extra precision (XP) mode. This mode recognizes the ligands forms that would possess unfavourable energies. The computational algorithm recognizes and provides a corresponding score based on the hydrophobic contact, interactions of hydrogen bonding. The glide G score was analyzed along with the bonding and non-bonding ligand–protein interactions and the type of exhibited interactions.

Similarly, the docking of FDA-approved compounds was done on the same grid that was used for l-NAT docking. All the ligands from FDA approved list were that were subjected to Ligprep were docked with NK1R via high throughput screening (HTVS). Then based on interactions and glide scores they were selected for standard precision (SP) studies. These candidates were further screened via extra precision (XP) studies. Since our focus was intended on the antagonistic actions on NK1R in the CNS, the FDA-approved drugs were screened for their ability to cross the blood–brain barrier (BBB). This was done by assessing the predicted BBB crossing ability through admetSAR software scored on a scale of 0–1. The scores in positive values and near to 1 depicted better BBB crossing abilities (https://dev.drugbank.com/guides/terms/blood-brain-barrier).

Therefore, among these candidates finally, the top 13 compounds were selected and ranked based on glide score, the number of bonding interactions and non-bonding interactions, MMGBSA energy, lipophilicity, hydrophobic enclosure reward, H-bond scores, electrostatic rewards, sitemap ligand/receptor non-H-bonding polar/hydrophobic and hydrophilic/ hydrophobic complementary terms, electrostatic rewards, rotatable bond penalty, and Epik state penalty scores. Out of these best 3 compounds were selected and subjected to molecular dynamic studies.

### Molecular dynamics (MD)

To create a simulation of the actual conditions in which the protein–ligand complex operates in the presence of solvents, membranes, and counter ions is essential along with the protein–ligand complex. This is performed using the Molecular Dynamic module of the Schrodinger Suite. When a system is subjected to these MD simulations, it creates its Newtonian dynamics followed by the creation of a trajectory pathway for the axis coordinates, speed, and even the energies of the particles in the system. The glide Gscores obtained from docking studies were compared and the best results were subjected to molecular dynamic (MD) studies using the Desmond System Builder module in Schrodinger software. Using the system builder, a predefined SPC solvent system was created using a boundary made with an orthorhombic box of 10 Å and 90° angles. Sodium and chloride ions at a concentration of 0.15 M were added to the system. Molecular Dynamics were run with a simulation time of 20 ns calculating about 1000 frames with NPT Ensemble class. The temperature of 300 K and pressure of 1.01 bar were maintained throughout the simulation. RESPA integrator was used of bonded time steps of 2 fs for which near was 2 and far was 6. The Nose Hoover chain thermostat method was used for a relation time of 1 ps. Martyna-Tobias-Klein barostat method was used for a relaxation time of 2 ps employing an isotropic coupling style. The cutoff short-range method at the cut-off length of 9 Å was employed. l-NAT and the top 3 selected compounds from XP docking studies of FDA-approved candidates were subjected to MD studies at 100 ns simulation time.

### Molecular mechanics with generalized born and surface area (MM-GBSA) studies

MM-GBSA studies combine calculations of molecular mechanics long with solvation models having a continuous system to generate the binding free energies of the complexes. The selected site and the ligand were subjected to binding energy studies using the Prime MM-GBSA module in Schrodinger Maestro software. The higher the negative value of the binding free energy of the complexed structure, the better the strength of the complex. The MMGBSA of l-NAT and FDA-approved compounds was performed and analyzed.

## Results and discussion

The contribution of Aβ and NFT has significantly impacted the progression of AD [24]. But recently, offering a synergistic role, the astrocytes, and microglia in the CNS have taken over the limelight. The activation of the microglial NK1R axis and subsequent release of superoxide and nitric oxide free radicals, and more importantly the pro-inflammatory mediators like IL-1, IL-6, and tumor necrosis factor-α (TNFα) has promoted neuroinflammation and neurodegeneration [8, 25, 26]. Thus, our in silico analysis of NK1R inhibitors could provide a kick start towards the successful management of neuroinflammation in AD.

### Prepared protein and ligand

The crystal structure of NK1R after being subjected to pre-processing steps was added with missing loops, rendered free of water molecules and side chains were filled. The pH of 7 was maintained and the energy was minimized by subjecting to the OPLS3e force field. The structure of NK1R is shown in Table 1 section a. The ligand l-NAT, after being subjected to ligand preparation was devoid of hydrogen, as seen in Table 1 section b. Similarly, the FDA-approved ligands were processed and made fit for further studies.

**Table 1:**
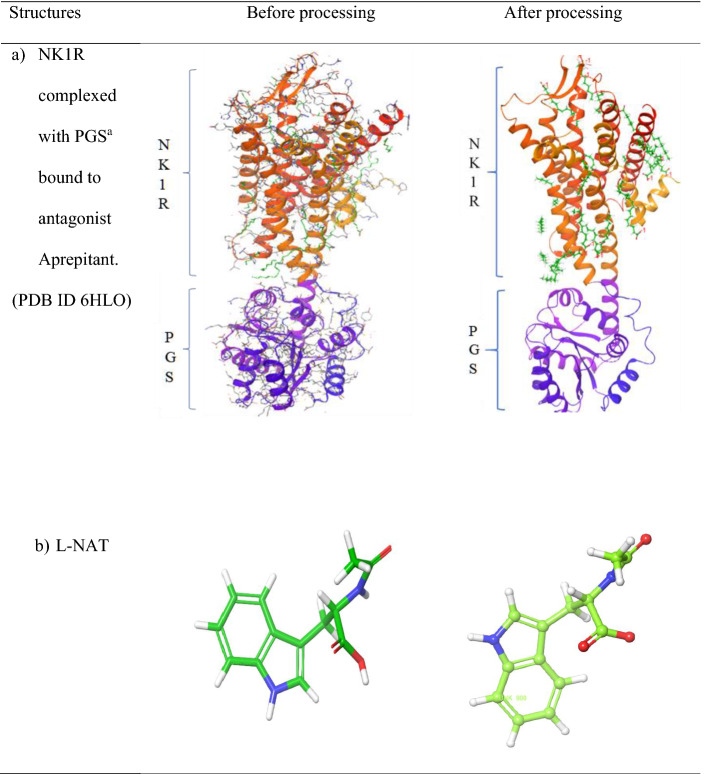
Crystal structures before and after processing

^a^*PGS* Pyrococcus abysii glycogen synthase domain

### Additional druggable sites found in NK1R

The structure of NK1R (PDB ID: 6HLO) obtained from PDB was co-crystallized with aprepitant, an antagonist whose location suggested the active site of the receptor. To confirm this notion, we carried out studies to identify possible druggable sites on the receptor using the Site Map module in Schrodinger Maestro. A total of 5 most druggable sites were obtained as shown in Table 2 and out of that top site (site 1) was selected based on site score and D score.

**Table 2:**
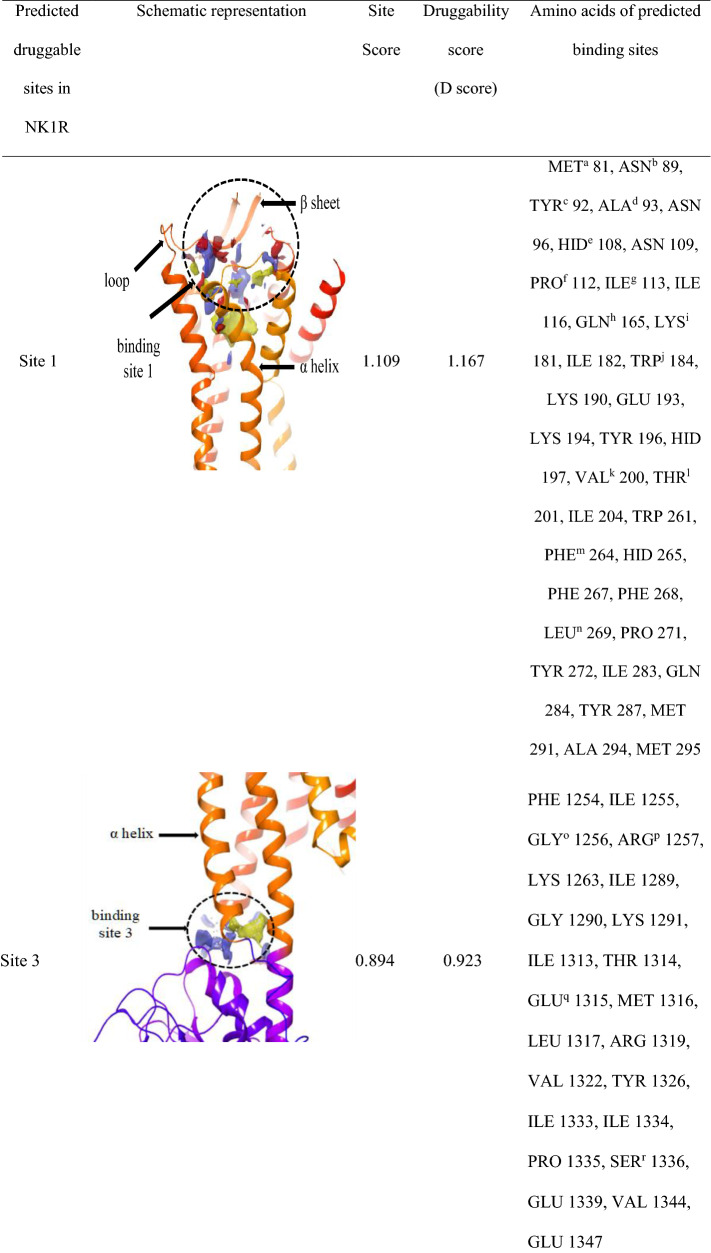

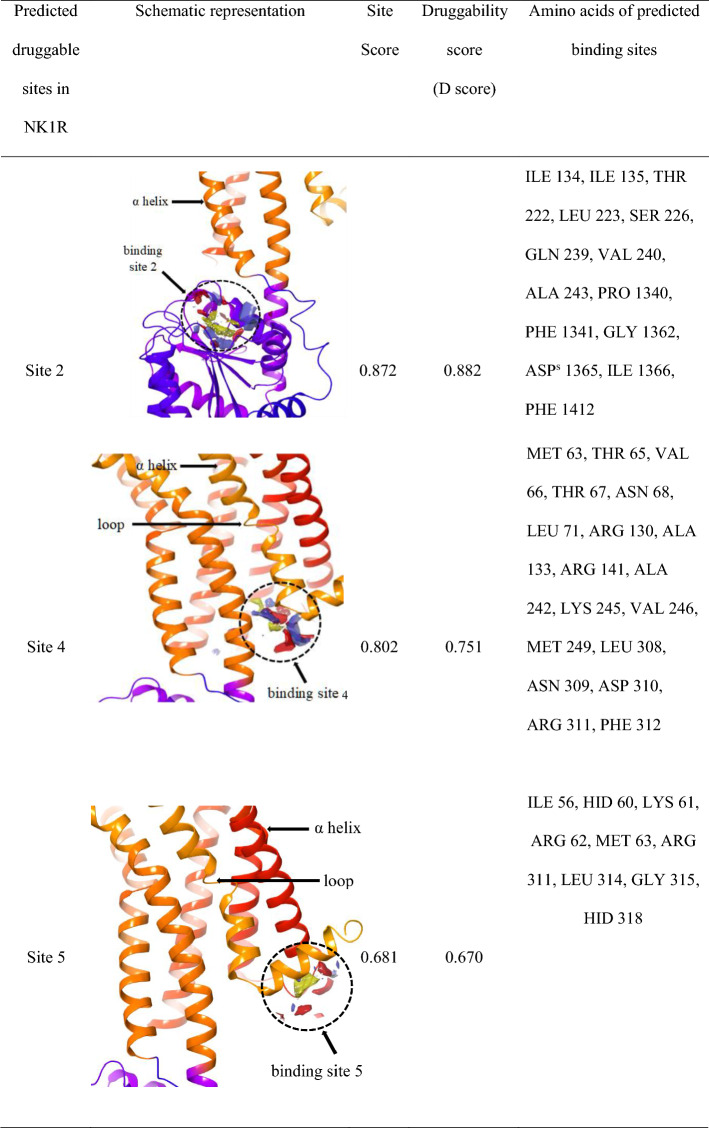
Predicted druggable sites in NK1R

^a^Methionine, ^b^Asparagine, ^c^Tyrosine, ^d^Alanine, ^e^Histidine, ^f^Proline, ^g^Isoleucine, ^h^Glutamine, ^I^Lysine, ^j^Tryptophan, ^k^Valine, ^l^Threonine, ^m^Phenylalanine, ^n^Leucine, ^o^Glycine, ^p^Arginine, ^q^Glutamate, ^r^Serine, ^s^Aspartate

The classification of binding sites is made through its D score where the sites above 1 are termed as druggable, while values ranging from 0.8 to 1 are intermediate druggable and values below 0.8 are non-druggable. Similarly, site score is used to compare the binding sites where values greater than 1 are considered ideal [27]. In our research, Site 1 showed a site score of 1.10, and a D score of 1.16 was selected for further studies. Interestingly, this was the same site as the one to which the aprepitant was seen to be bound. Our SiteMap analysis showed four other additional binding sites with site scores and D scores less than 1 that indicated the presence of a single prevalent binding site of NK1R that is used by the antagonist in the real world scenarios.

### Grid generation

**Fig. 3:**
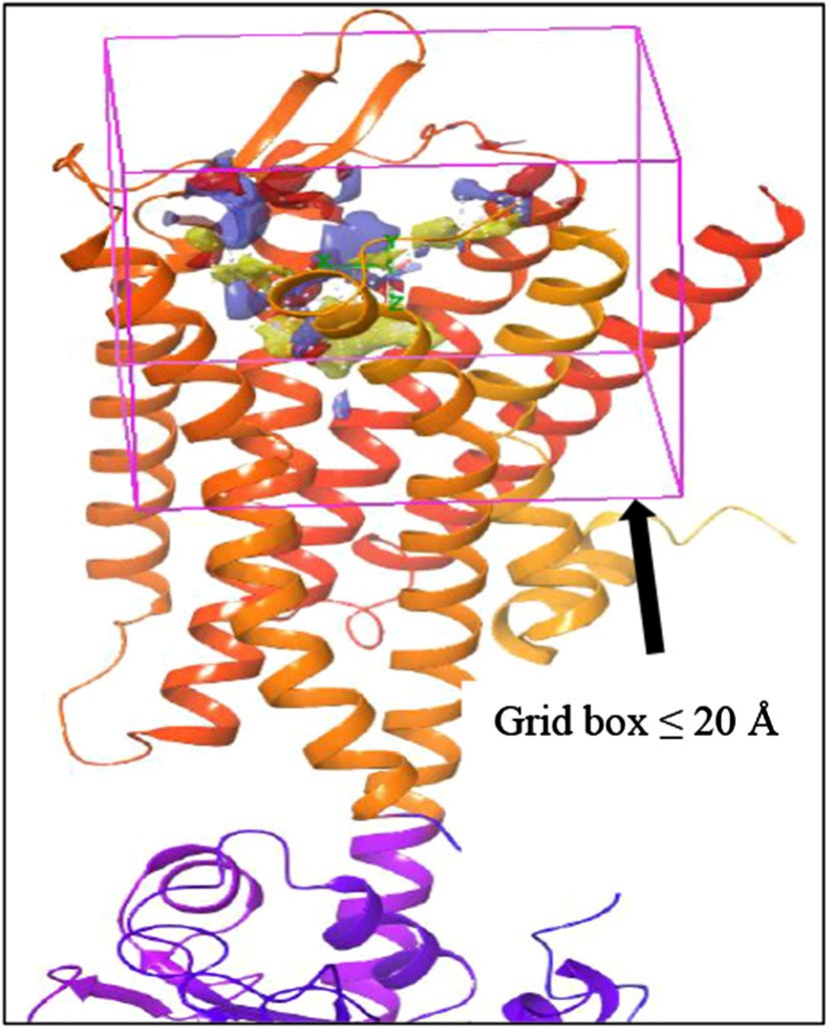
Grid Generation around the Site 1

The receptor grid generation module of the Schrodinger suite generated a grid around the selected atoms within the area of the site predicted by SiteMap. As seen in Fig. 3 a grid was made with a grid box in the center of selected atoms with ligand length ≤ 20 Å that provided good specificity to the region of ligand binding. The length of the grid box can be adjusted as per the sites. A maximum value of 50 Å is possible while very low values can restrict the binding capabilities to smaller regions and focus it on the binding sites. Still, it must be ensured that the entire site is encapsulated into the box. Usually, the site location generated by the SiteMap may be scattered over a region so a smaller region of the grid box can be selected therefore we chose a value of ≤ 20 Å. This was further selected for ligand docking studies.

#### Molecular docking studies with NK1R

**Fig. 4:**
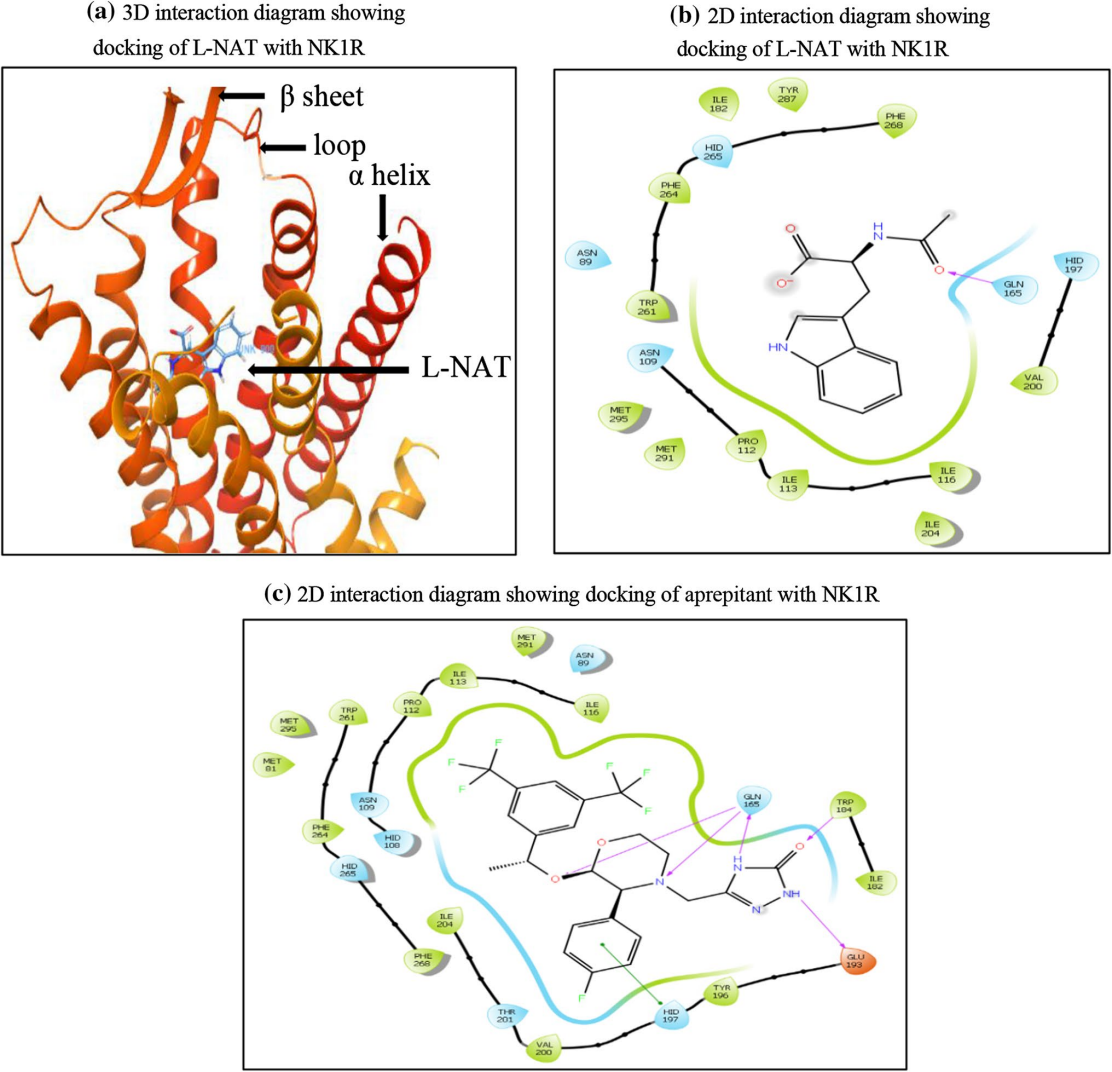
**a** 3D interaction diagram showing docking of l-NAT with NK1R. **b** 2D interaction diagram showing docking of l-NAT with NK1R. **c** 2D interaction diagram showing docking of aprepitant with NK1R

Site 1 was subjected to docking studies based on the D score and site score obtained earlier. Glide Ligand docking module was used to perform docking studies in XP mode. The glide score separates the compounds with high binding affinity to intermediate and no binding affinity. The 3D interaction of L-NAT with NK1R is shown in Fig. 4a. The orthosteric binding pocket of NK1R expresses amino acids namely Glutamine (GLN) 165, Glutamate (GLU) 193, Histidine (HIS) 197, Tyrosine (TYR) 272, Phenylalanine (PHE) 268, PHE 264, Aspartate (ASN) 109, Isoleucine (ILE) 113, Proline (PRO) 112, ILE 204, Threonine (THR) 201 and HIS 265 [28–30]. One of the most important amino acids needed for antagonistic activity of NK1R, GLU 165 was seen to be interacting with l-NAT as seen in Fig. 4b. A docking score of − 7.652 kcal/mol was obtained predicting the desired antagonistic property of l-NAT with NK1R.

Aprepitant has been approved as a standard NK1R antagonist to treat chemotherapy-induced nausea and vomiting (CINV) [31]. It showed a docking score of − 15.5 kcal/mol. Therefore, we considered aprepitant as the lead molecule to identify the interaction sites with the amino acid sequence for l-NAT and FDA approved ligands. As shown in Fig. 4c below, the interaction of aprepitant that is a standard inhibitor for NK1R forms a good hydrophobic pocket showing three hydrogen bonds with GLN 165, 1 hydrogen bond with TRP 184, and 1 hydrogen bond with GLU 193, and pi-pi stack with HIS 197. Most of these interactions form a part of the essential interactions needed for the antagonistic activity of NK1R.

A total of 2115 FDA-approved compounds were subjected to molecular docking studies with NK1R in HTVS mode. These studies predicted 2039 compounds based on their glide gscore which were then selected for docking studies in SP mode. The SP docking studies nearly predicted 100 candidates again based on glide gscore which were subsequently subjected to XP docking studies that showed 55 possible candidates for NK1R inhibition. Further, we selected the candidates having glide gscores up to 7.0 kcal/mol. Since our main aim was to target the NK1R of the CNS, upon segregating these compounds for their ability to cross BBB we finally shortlisted the top 13 compounds.

The analysis of l-NAT as shown in Table 3 and the top 13 FDA approved compounds as shown in Table 4 were screened for parameters like glide gscore, the number of bonding interactions and non-bonding interactions, ChemScore lipophilic pair term and the fraction of the total protein–ligand vdW energy, Hydrophobic enclosure reward, ChemScore H-bond pair term, Electrostatic rewards, SiteMap ligand/receptor non-H-bonding polar/hydrophobic and hydrophilic/hydrophobic complementary terms, Rotatable bond penalty, Epik State Penalty and MMGBSA scores showed their ability to interact with NK1R.

The XP docking of l-NAT with NK1R revealed a low MM-GBSA score of − 18.81 kcal/mol suggesting fair stability of the ligand-receptor complex as compared to MM-GBSA score of − 93.09 kcal/mol of aprepitant. The ChemScore lipophilic pair term and the fraction of the total protein–ligand vdW energy of − 4.07 were good enough to depict the lipophilic character of the complex along with the hydrophobic enclosure reward of − 1.48. The hydrogen bonding within the protein–ligand complex was strong with an H-bond value of − 0.35. A very low electrostatic reward value of − 0.08 denoted negligible instabilities due to electrostatic interactions. The ligand was stable into the orthosteric pocket as shown by the SiteMap score of − 0.41. A low rotatable bond penalty of 0.25 and EpikState penalty of 0 showed that less amount of faulty bonds were involved in the protein–ligand complex as shown in Table 3. This suggested the possibility of l-NAT as an NK1R inhibitor.

**Table 3:**
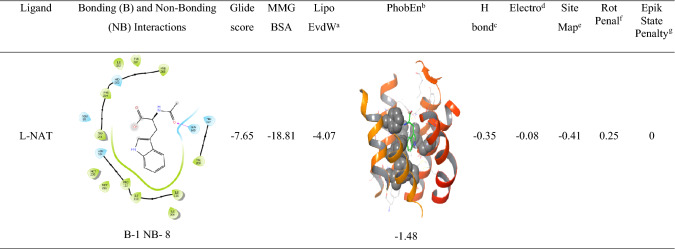
XP docking results of l-NAT with NK1R

^a^ChemScore lipophilic pair term and fraction of the total protein ligand vdW energy

^b^Hydrophobic enclosure reward

^c^ChemScore H-bond pair term

^d^Electrostatic rewards

^e^SiteMap ligand/receptor non H-bonding polar/hydrophobic and hydrophilic/hydrophobic complementary terms

^f^Rotatable bond penalty

^g^EpikState Penalty

Analysis of indacaterol revealed 4 bonding interactions namely pi-pi stack and hydrogen bonding with HIS 197, pi-pi stack with PHE 268, and a hydrogen bonding interaction with ASN 109. These amino acids constitute the essential interactions required for NK1R antagonism. The interactions yielded a glide gscore of − 7.42 kcal/mol along with binding free energy MMGBSA energy of − 58.55 kcal/mol. The druggable site of the protein was having excellent lipophilic surroundings due to which it showed ChemScore lipophilic pair term and fraction of the total protein–ligand vdW energy of − 6.62 kcal/mol and hydrophobic enclosure reward of − 2.23 kcal/mol. The hydrogen-bonding interactions present in the system showed a high amount of stability evident by the high H bond score of − 1.06 as shown in Table 4. The electrostatic reward score of − 0.54 was obtained. Surprisingly, the sitemap score was 0 predicting some instability in the protein complex. The lower rotatable bond penalty of 0.22 and nil EpikState penalty served as an advantage for favouring antagonistic action to NK1R.

**Table 4:**
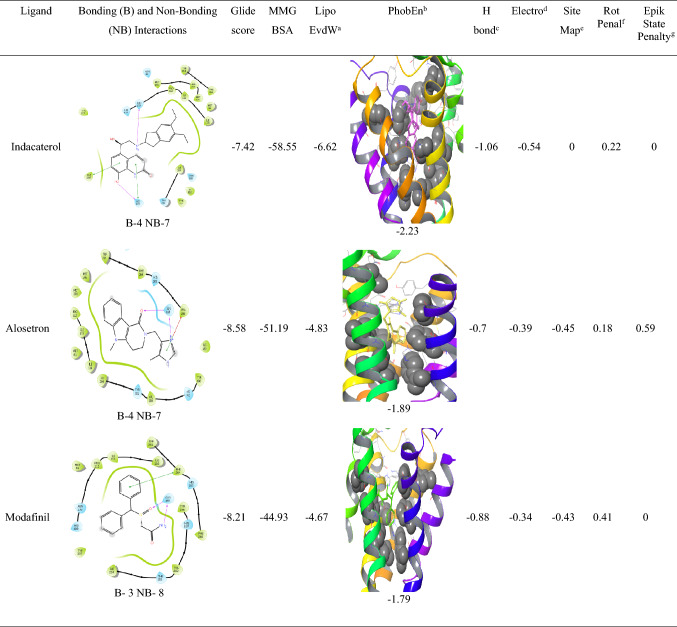

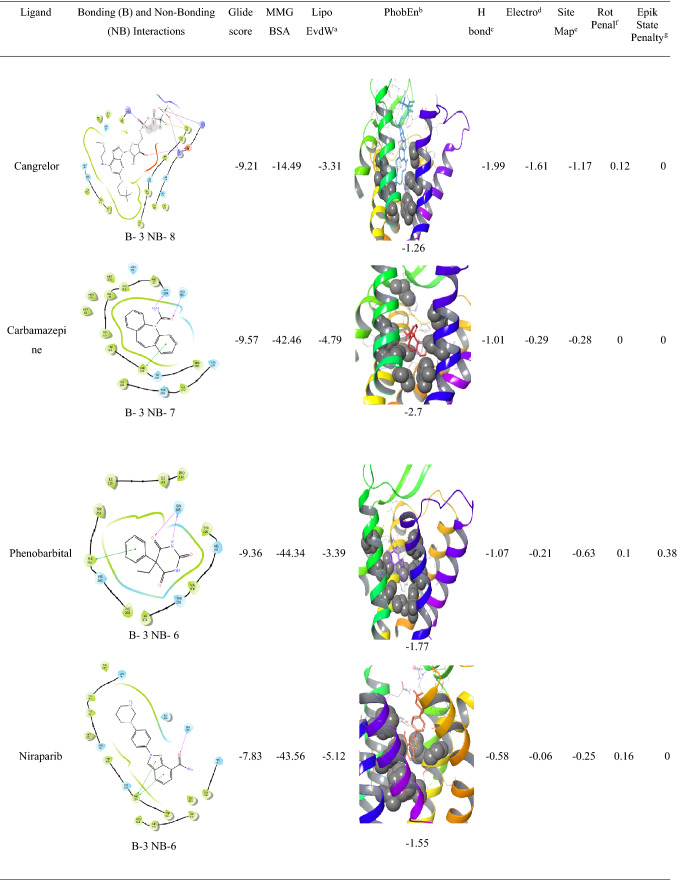

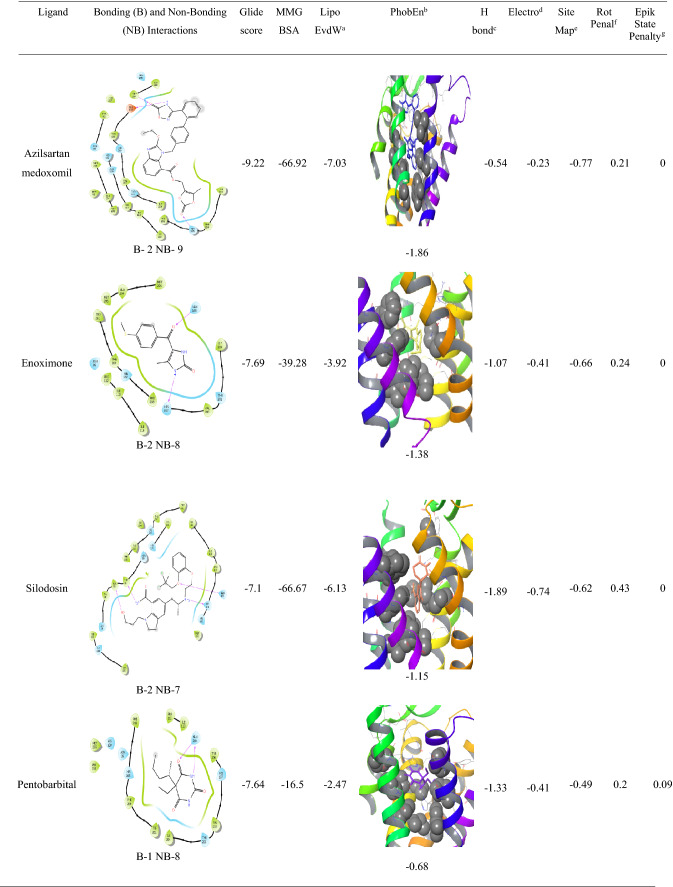

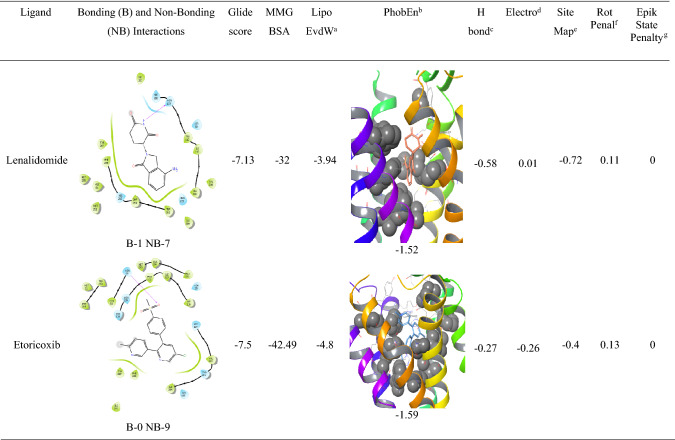
XP docking results of top 13 FDA-approved candidates with NK1R

^a^ChemScore lipophilic pair term and fraction of the total protein ligand vdW energy

^b^Hydrophobic enclosure reward

^c^ChemScore H-bond pair term

^d^Electrostatic rewards

^e^SiteMap ligand/receptor non H-bonding polar/hydrophobic and hydrophilic/hydrophobic complementary terms

^f^Rotatable bond penalty

^g^EpikState Penalty

Alosetron showed 4 bonding interactions namely pi-pi stack and pi cation with PHE 268 and 2 hydrogen bonding interactions with GLN 165. It also showed 7 non-bonding interactions with multiple amino acids of the allosteric binding pocket of NK1R as evident in Table 4. Its glide gscore of − 8.58 kcal/mol showing great binding ability with NK1R was supported by its binding free energy MMGBSA energy of − 51.19 kcal/mol. The ChemScore lipophilic pair term and the fraction of the total protein–ligand vdW energy of − 4.83 and hydrophobic enclosure reward of − 1.89 signified denoted good lipophilic characteristics of the complex. The H-bond score of − 0.7 suggested significantly high energy of the hydrogen bonding with the protein–ligand complex. The protein–ligand complex was stable in the system due to a low electrostatic reward value of − 0.39. The sitemap score of − 0.43 implied good stability of the ligand into the orthosteric pocket of NK1R. A low rotatable bond penalty of − 0.43 and Epik State penalty of 0 as shown in Table 4 showed that less amount of faulty bonds were involved in the protein–ligand complex suggesting good capability of alosetron as NK1R inhibitor.

Modafinil was ranked third in our analysis due to the 3 bonding interactions namely pi-pi stack with PHE 264 and 2 hydrogen bonding interactions with GLN 165 and 7 nonbonding interactions. Interestingly, as evident in Table 4`, it could be seen that Cangrelor also had a similar number of bonding and nonbonding interactions, but it was not given preference over modafinil since the latter showed interactions with important amino acids (PHE 264 and GLN 165) as compared to earlier (GLU 193 and TYR 272) and also mainly since important interactions with GLN 165 and HIS 197 were missing. Another reason was the higher ChemScore lipophilic pair term and fraction of the total protein–ligand vdW energy of − 4.67 kcal/mol compared to − 3.31 kcal/mol of cangrelor. Modafinil showed a hydrophobic enclosure reward score of − 1.79 kcal/mol and an H-bond score of − 0.88 kcal/mol. It showed a low electrostatic reward score of − 0.34 hinting at less unwanted electrostatic interactions as shown in Table 4. Interestingly, the rotatable bond penalty was as low as 0.41with nil Epik state penalty suggesting favourable properties to bind with NK1R promoting its antagonism.

### Good, bad and ugly interactions

Based on the distance of atoms and their van der Waals radii, the van der Waals interactions are termed as good interactions, bad interactions, and ugly interactions [32]. Mathematically, the contact types are categorized based on ratio calculated using the formula $C=D12/\left(R1+R2\right) ,$ where C is the type of contact, D12 denotes the distance between atoms 1 and 2, and R1 R2 are the corresponding van der Waals radii of both atoms 1 and 2, respectively. The ideal range for good interactions lies from 1.30 to 0.89; while bad interactions range from 0.89 to 0.75 and ugly interactions produced values lesser than 0.75 [33]. Therefore, the higher the good interactions with the required atom, the better binding characteristics are shown by the complex. l-NAT was able to produce 5 good interactions with atoms interacting with target amino acid while aprepitant produced 6 good interactions. The good interactions show important bonds that are essential in binding to the receptor. On the contrary, it was worth noting that l-NAT showed 2 bad interactions while aprepitant showed 3 bad interactions. l-NAT showed no ugly interactions while the aprepitant showed one ugly interaction as shown in Table 5. The bad interactions and ugly interactions are undesirable ones.

**Table 5:**
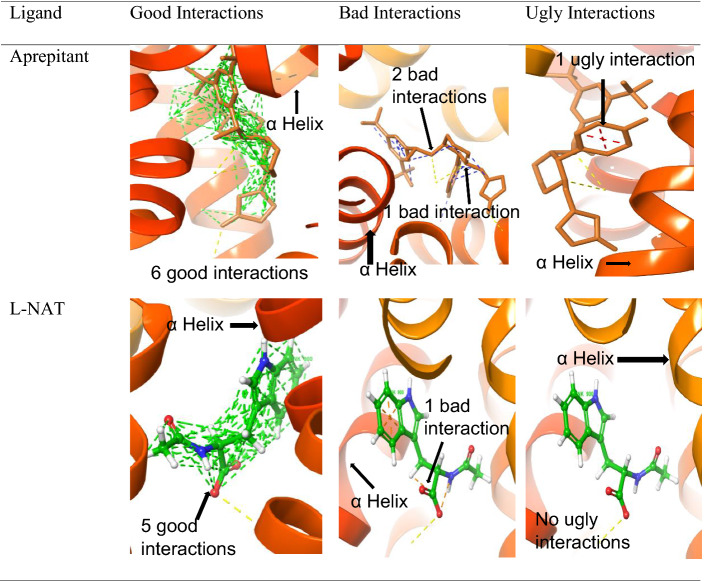
Comparison of good, bad, and ugly interactions shown by aprepitant and l-NAT

Similarly, alosetron and indacaterol showed good interactions and bad interactions with NK1R as shown in Table 6. Alosetron showed 5 good interactions along with the hydrogen non-covalent bonds and 18 good interactions along with pi-pi stacking and pi cation bonds. Whereas indacaterol showed 5 good interactions along with the hydrogen non-covalent bonds and 29 good interactions along with pi-pi stacking bonds.

**Table 6:**
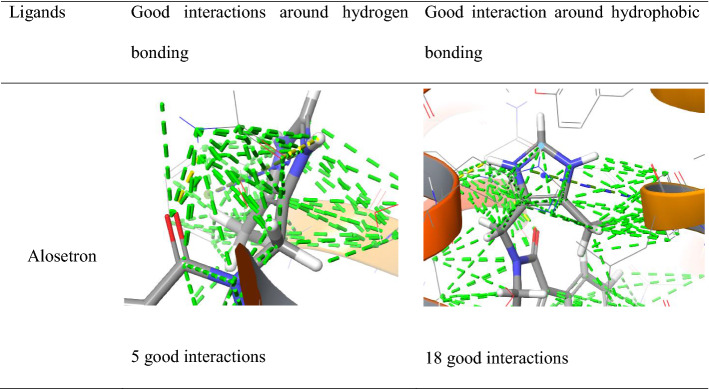
Good interactions depicted by Alosetron and Indacaterol from docking with NK1R

Along with good interactions, alosetron and indacaterol showed bad interactions as shown in Table 7. But were devoid of any ugly interactions. Alosetron and indacaterol showed 2 bad interactions.

**Table 7:**
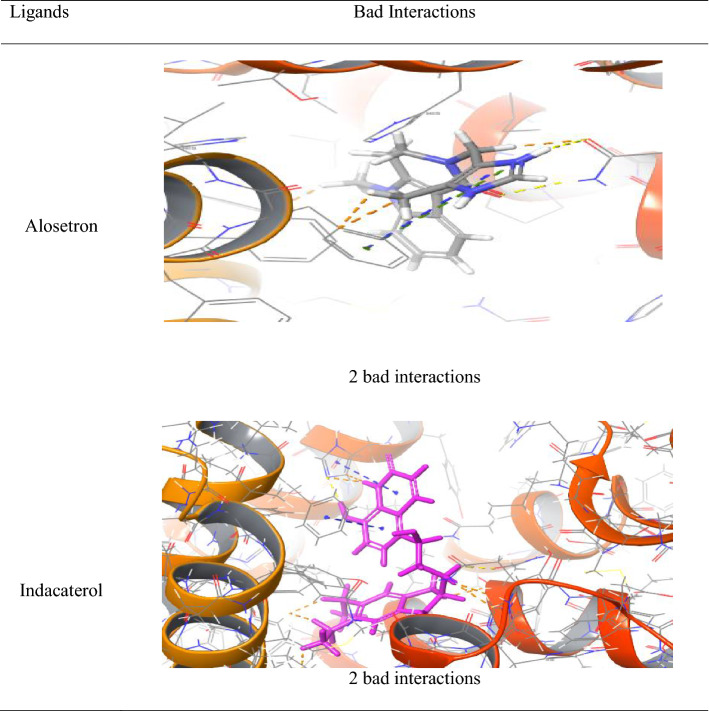
Bad interaction shown by alosetron and indacaterol

### Molecular dynamic (MD) studies

Molecular Dynamic Studies carried out on l-NAT, alosetron, modafinil and indacaterol provided detailed insight into the stability of protein–ligand interaction in a solvent system.

#### Protein–ligand root mean square deviation (RMSD)

RMSD is a parameter known to measure a relative change in the displacement of atoms for a given frame with regard to a reference frame. A total of 1000 frames were captured in a period of 100 ns. The formula used to calculate the RMSD is shown in Table 8.

**Table 8:** Formula for the calculation of RMSD

RMSD formula	Symbol	Explanation
$\mathbf{R}\mathbf{M}\mathbf{S}\mathbf{D}\mathbf{x} =\frac{1}{{\varvec{N}}}{\sum }_{{\varvec{i}}=0}^{{\varvec{N}}}\boldsymbol{ }\{\boldsymbol{ }\left[{{\boldsymbol{ }{\varvec{r}}}^{\boldsymbol{^{\prime}}}}_{{\varvec{i}}}\left({\varvec{t}}{\varvec{x}}\right)\right]-{[{\varvec{r}}}_{{\varvec{i}}}({{\varvec{t}}}_{{\varvec{r}}{\varvec{e}}{\varvec{f}}})]{\boldsymbol{ }\}}^{2}$	N	No. of atoms in the selection of atoms
	${t}_{ref}$	Reference Time, where initial frame = reference frame and time *t* = 0
	${r}^{^{\prime}}$	Position of selected atoms in x frame, post superimposition on the reference frame, provided the frame x is captured at time *t*_x_

**Fig. 5:**
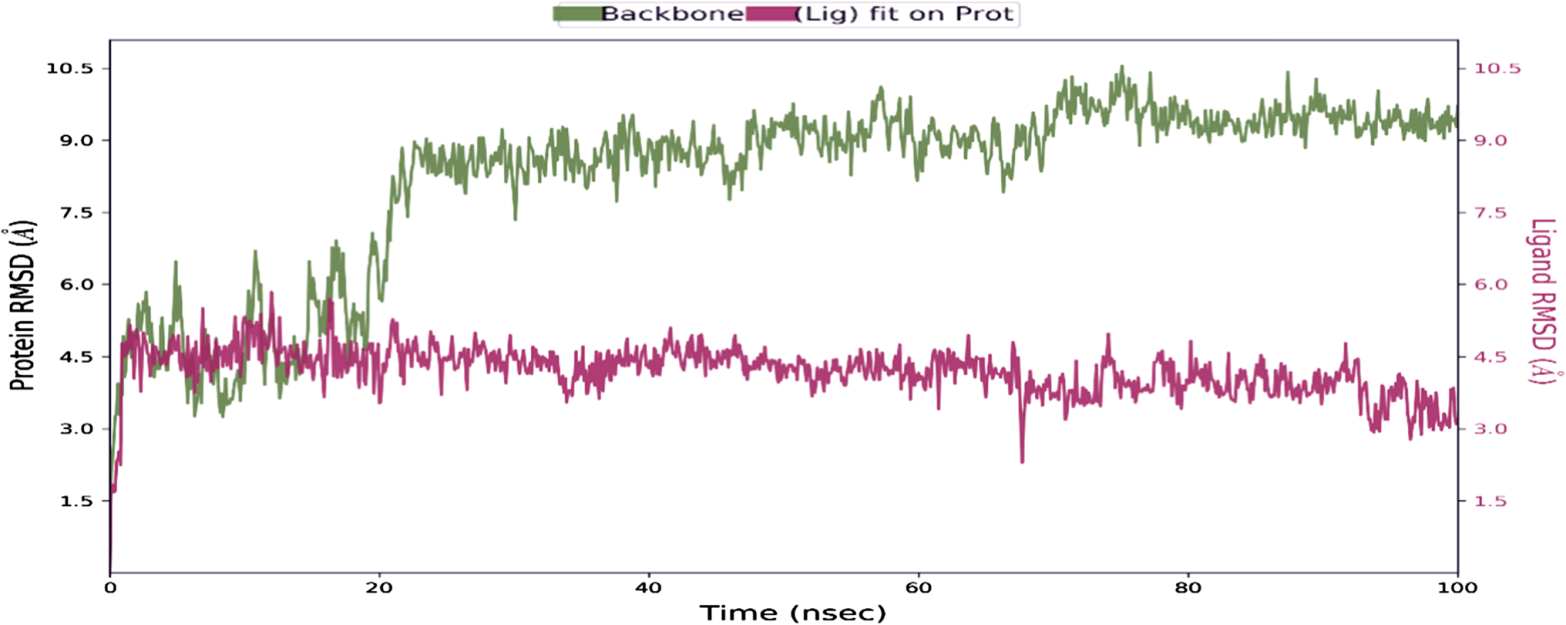
The RMSD obtained on the interaction of l-NAT with NK1R ran for 100 ns

Ideally, for smaller proteins, the protein RMSD fluctuations of around 1–3 Å are accepted. In our results, we saw a significant variation in protein RMSD values. The interaction of l-NAT with NK1R for a period of 100 ns showed that l-NAT was stable for a period of 20 ns but later on due to changes in structural conformations it was seen to move out of orthosteric binding pocket as evident in Fig. 5. The ligand RMSD was stable as compared to the protein RMSD. The ligand is seen to stabilize nearly at 4.5 Å but the protein is highly unstable has attained an RMSD value up to nearly 10.5 Å. During the initial 10 ns, the ligand and protein were in continuous contact and thus the complex showed the best stability. The RMSD for protein shown in green is seen to undergo a huge conformational change beyond 20 ns while the RMSD for the ligand is shown in red. The ligand RMSD shows the stability of the ligand corresponding to the binding site and the receptor. In the initial phase, the ligand RMSD was found to be closer to protein RMSD that showed that ligand was still in proximity, but later, the higher difference in the ligand RSMD and protein RMSD could suggest the movement of ligand away from the binding site.

The RMSD for alosetron was nearly 5 Å while NK1R protein attained values of up to 9 Å as shown in Table 9. Even though the RMSD values appear to be high, there is significant contact between the ligand-receptor complex indicating a good interaction. Similarly, indacaterol also showed RMSD of up to 4 Å and NK1R exhibited values up to 8 Å but it could imply good stability of the complex. Modafinil was seen to exhibit RMSD fluctuations of nearly 5 Å during the 10–15 ns simulation time but was later seemed to be stabilized at 2.5 Å throughout the remaining time while NK1R showed RMSD of 6.5 Å. Good contact was observed between modafinil and NK1R.

**Table 9:**
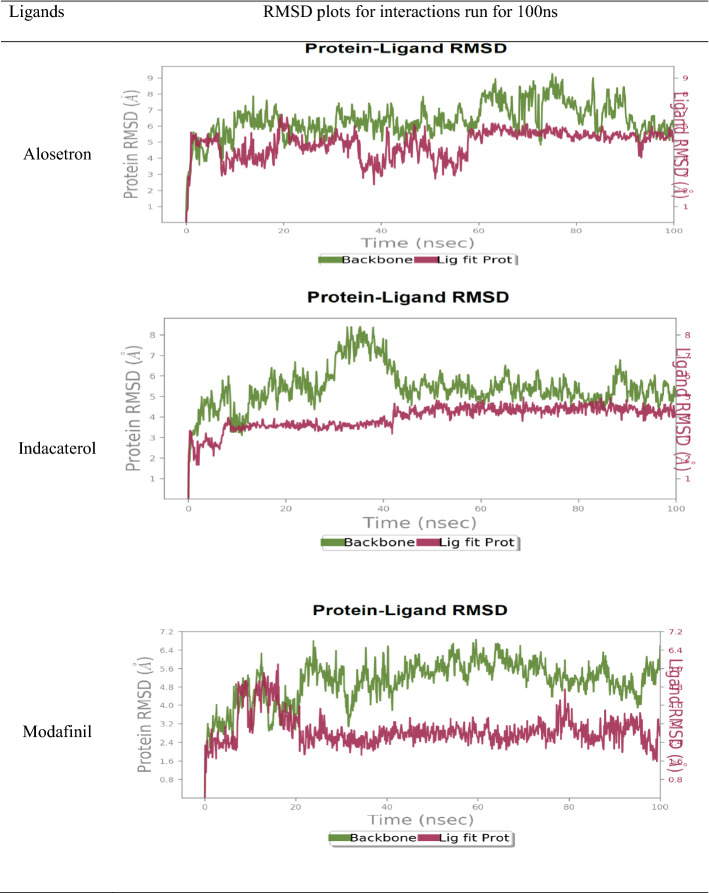
The RMSD plots of alosetron, indacaterol, and modafinil for simulations run for 100 ns

#### The protein–ligand and ligand–protein contacts

Hydrogen bonds impose a strong influence on the adsorption, metabolism, and specificity of the receptor. They can be either backbone acceptor or backbone donor or can be side-chain acceptor or side-chain donors. Hydrophobic contacts include interactions of the hydrophobic amino acids and an aromatic or aliphatic moiety of the ligand. Water bridges are formed when the ligand interacts with the protein through interactions facilitated by a water molecule. Ionic interactions occur between two oppositely charged atoms within the vicinity of 3.7 Å of each other and contain no H bonds.

**Fig. 6:**
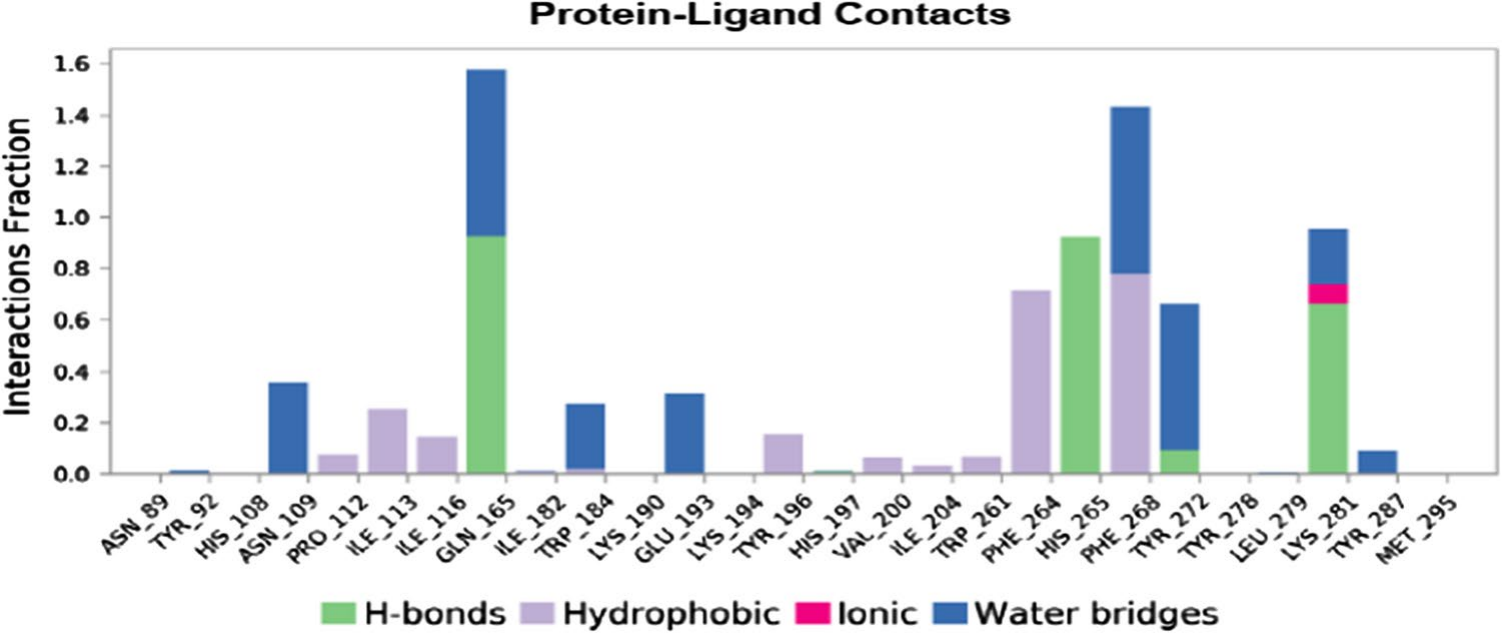
Protein–ligand contacts through hydrogen bonds, hydrophobic bonds, ionic bonds, and water bridges exhibited by l-NAT in complex with NK1R

In the interaction of l-NAT with NK1R, even though the complex seemed to drift apart beyond the 20 ns phase of MD probably due to high conformational change in the protein, there were multiple types of bonds formed with amino acids of the protein namely hydrogen bonds, hydrophobic and water bridge bonds as shown in Fig. 6.

The FDA-approved ligands namely alosetron, indacaterol, modafinil showed hydrogen bonding, hydrophobic contacts, and water bridges with desired amino acids of NK1R but surprisingly Niraparib failed to show even a single desired interaction with necessary amino acids. The protein–ligand contact maps have been shown in Table 10 and the details of amino acids with respective contacts have been elaborated in Table 11.

**Table 10:**
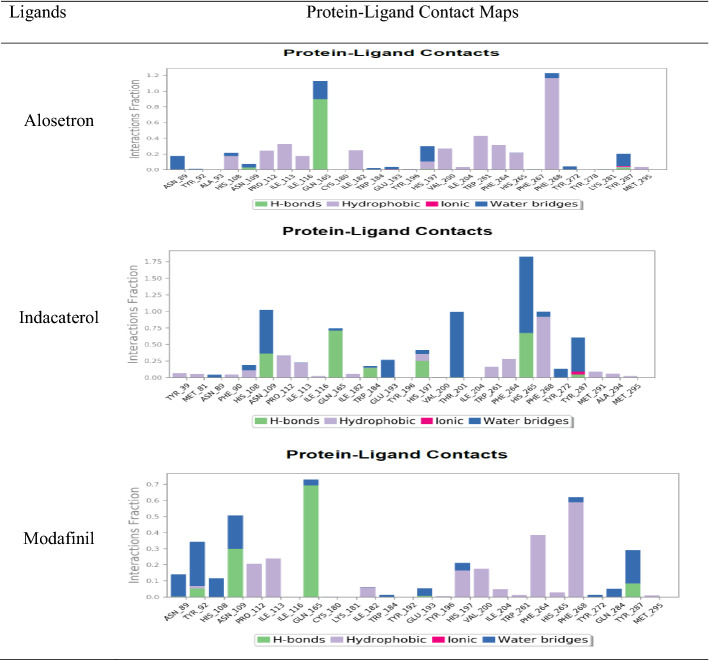
Protein–ligand contact maps of alosetron, indacaterol, and modafinil

**Table 11:** Summary of type of contacts shown by amino acids of NK1R with l-NAT, alosetron, indacaterol, and modafinil

Ligand	Type of contacts formed by amino acids needed for antagonistic activity of NK1R		
	Hydrogen bonds	Hydrophobic contacts	Water bridges
l-NAT	GLN 165, His 197, TYR 287, PRO 112	PHE 264, PRO 112, ILE 113 ILE 116, TRP 261, MET 291, and MET 295	PHE 264, GLN 165, His 197, TYR 287, ASN 89, HIS 108, ASN 109, PRO 112
Alosetron	ASN 109	His 197, ILE 204, PHE 264, PHE 268	ASN 109, GLN 193, His 197, PHE 268, TYR 272
Indacaterol	ASN 109, GLN 165, HIS 197	PRO 112, ILE 113, HIS 197, PHE 264, HIS 265, PHE 268	ASN 109, GLN 165, GLU 193, HIS 197, THR 201, HIS 265, PHE 268, TYR 272
Modafinil	ASN 109, GLN 165, GLU 193	PRO 112, ILE 113, HIS 197, ILE 204, PHE 264, PHE 268	ASN 109, GLN 165, GLU 193, HIS 197, PHE 268, TYR 272

**Fig. 7:**
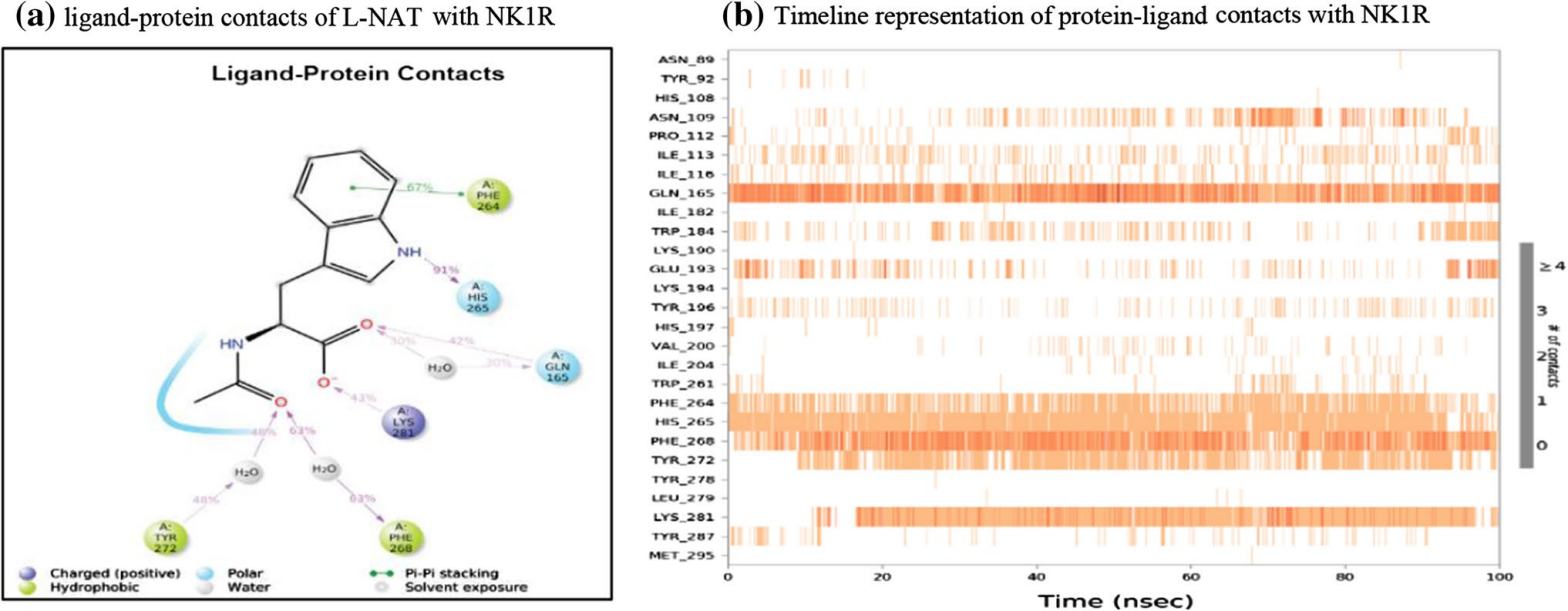
The L-NAT and NK1R contacts in 2D form and timeline representation

In the ligand–protein contacts exhibited by l-NAT with NK1R, PHE 264 shows pi-pi stack hydrophobic interaction with benzene ring of pyrrole for 67% of the simulation time. The oxygen of the carboxylate group interacts with GLN 165 for nearly 42% of the time while it also forms an interaction via water bridge through the water molecules present in the solvent system, for 30% of the simulation. Similar water bridges are seen with PHE 268 and TYR 272 for 63% and 48% of the simulation as shown in Fig. 7a. Therefore, the existence of these interactions could signify good antagonistic properties of l-NAT.

Figure 7b shows the binding affinity of the amino acid interactions in the form of a timeline pattern. It shows the contacts made by the ligand with specific amino acids of the protein. Sometimes, more than one specific contact is made by ligands due to which the bands appear darker. Multiple amino acids of the protein have shown contacts with l-NAT and more importantly, ASN 109, GLN 165, HIS 197, THR 201, PHE 264, TYR 287 showed strong binding affinity. The consistent dark bands indicate the strength of the binding signifying the antagonistic activity of l-NAT.

**Fig. 8:**
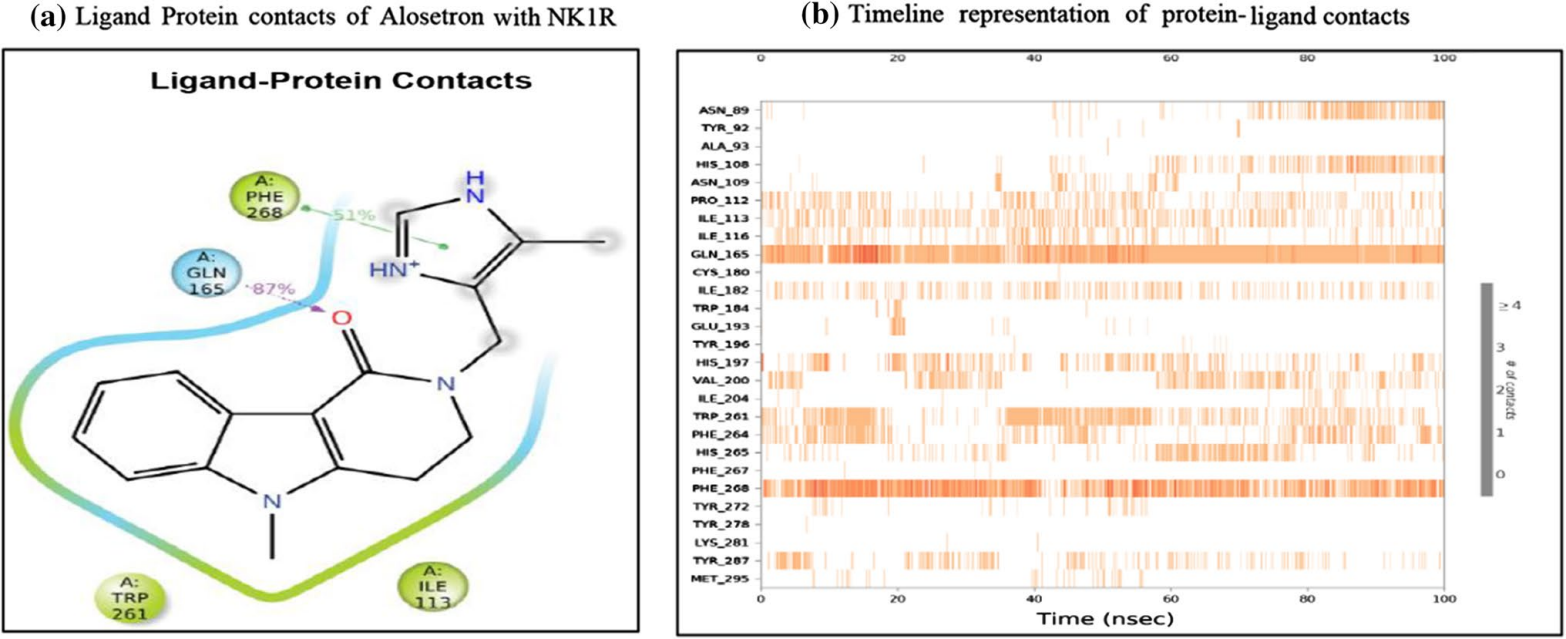
The Alosetron and NK1R contacts in 2D form and timeline representation

The PHE 268 forms a pi-pi stack with an imidazole ring of alosetron for nearly half of the simulation period while GLN 165 forms a hydrogen bond with the carboxylic group for 87% of the time as shown in Fig. 8a. These interactions are very strong interactions and crucial in providing antagonistic activity to alosetron towards NK1R. Similarly, Fig. 8b shows contact in the form of timeline representation.

**Fig. 9:**
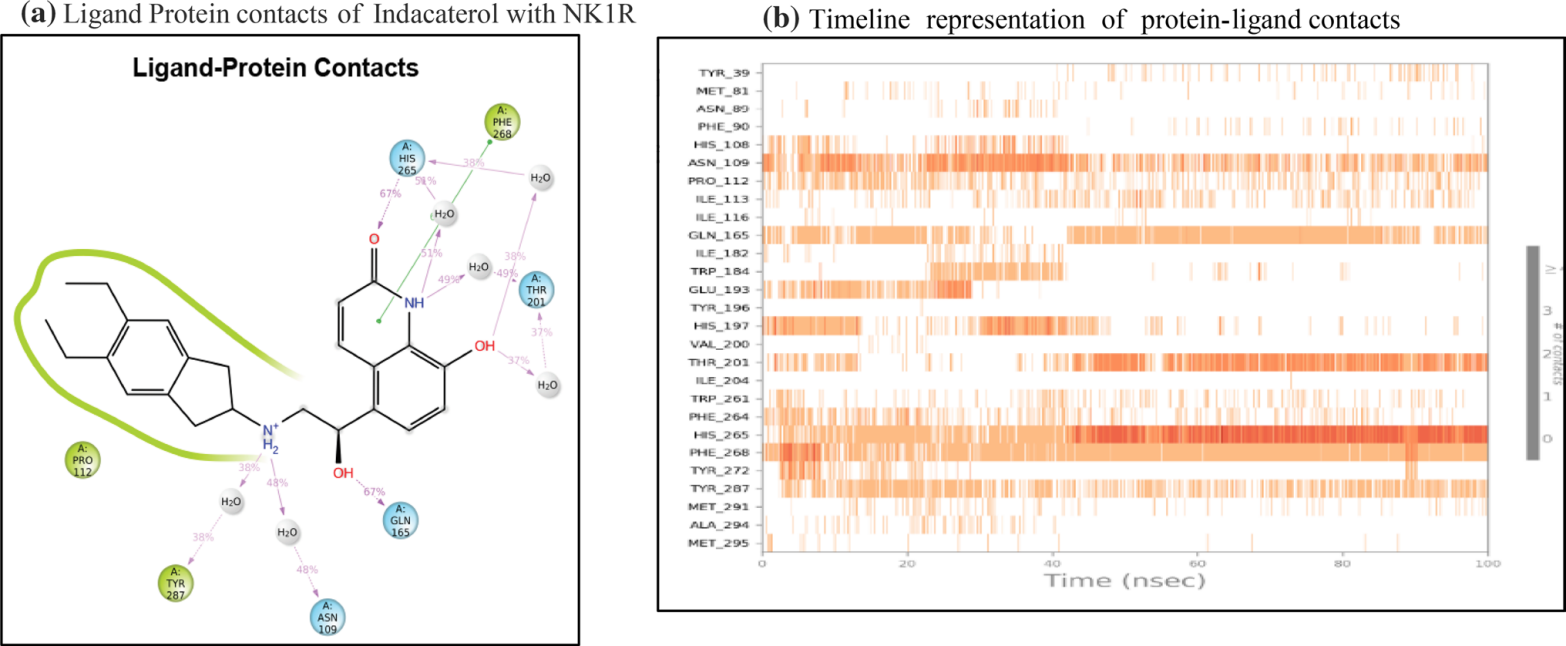
The Indacaterol and NK1R contacts in 2D form and timeline representation

In indacaterol, PHE 268 forms a pi-pi stack for 38% of the simulation time, whereas the -NH-C=O group interacts with the water molecules of the solvent system for nearly 50% of the simulation time. THR 201 forms a water bridge with the OH group of indacaterol with the help of solvent molecules 37% of the time. GLN 165 directly interacts with the OH group to form H bonds that are connected for nearly 67% of the time as shown in Fig. 9a. These interactions suggest that indacaterol can sustain several critical interactions needed for antagonistic activity with NK1R. These interactions are also depicted in timeline form as seen in Fig. 9b.

**Fig. 10:**
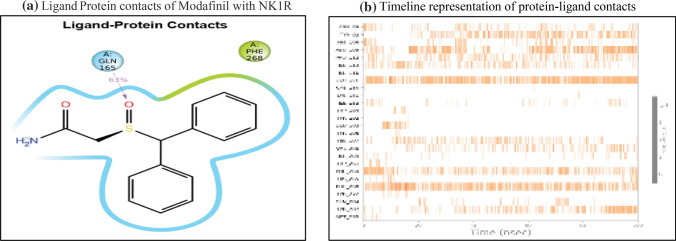
The Modafinil and NK1R contacts in 2D form and timeline representation

The S=O group in modafinil interacts with GLN 165 to form a hydrogen bond for nearly 63% of the simulation time as shown in Fig. 10a. As discussed earlier, GLN 165 is an important amino acid needed to show effective antagonistic activity and this interaction could favor the same. Apart from GLN 165, it also shows contacts with other amino acids as depicted in Fig. 10b in the form of a timeline.

The structural activity relationships reveal that the various chemical moieties present in the studied antagonists, interact with important amino acids of NK1R orthosteric binding pocket that are necessary to produce the antagonistic effect. As evident in our analysis, the acetate anion present in l-NAT is essential in interacting with the GLN 165, followed by the interaction of indole’s aromatic ring with PHE 268 and the indole’s amino group with HIS 265.

In indacaterol, the 8-hydroxyquinoline-2-one forms an important moiety in encouraging its antagonistic activity. The carbonyl group in this moiety interacts with HIS 265, while the substituted pyridine ring interacts with PHE 268. Another important moiety is the hydroxyethyl group responsible to interact directly with GLN 165. The alosetron contains a substituted pyrido indole group with moieties like carboxyl and amino groups. The carboxyl group confers important antagonistic activity due to its interaction with GLN 165 along with the imidazole ring that interacts with PHE 268. In modafinil, an important sulfoxide group that bridges dimethyl phenyl and acetamide group interact with GLN 165, a crucial amino acid responsible in promoting antagonistic activity in NK1R.

## Conclusion

Currently, the pharmacotherapy available against AD provides only symptomatic relief and our approach to target the root cause i.e., neuroinflammation via NK1R system that leads to neurodegeneration may be beneficial in AD. Our study showed that l-NAT, alosetron, indacaterol, and modafinil were able to show bonding and nonbonding interactions with the different amino acids of the NK1R orthosteric binding pocket namely GLN 165, GLU 193, HIS 197, TYR 272, PHE 268, PHE 264, ASN 109, ILE 113, PRO 112, ILE 204, THR 201 and HIS 265 GLN 165, HIS 197 in XP docking studies necessary for the inhibitory action of NK1R. The binding energy MMGBSA scores were also suggestive of good stability of the protein–ligand complex. Further, the interactions between the protein and ligand were found to be strong throughout 100 ns in MD studies showing good stability. The hydrophobicity, electrostatic interactions, and the inter ligand penalty was found to be good in terms of the ligand stability within the protein. The l-NAT, alosetron, and indacaterol showed many good interactions and few bad interactions favoring good binding characteristics. Overall, l-NAT, alosetron indacaterol, and modafinil showed potent abilities to interact with the amino acids of NK1R to facilitate its inhibition. Further in vitro and in vivo studies can be done to explore the mechanism of action of these molecules in inhibiting NK1R in detail that could be beneficial as a new therapeutic indication in the conditions of AD.

## Data Availability

The study was done and analyzed in Manipal—Schrödinger Centre for Molecular Simulations at Manipal College of Pharmaceutical Sciences, Manipal, Karnataka.

## Code availability

The study has been performed using a commercial computer-aided drug design software Maestro (Schrodinger Inc) Version 11.8.
